# Supplementary material for: Flux Calculation for Primary Metabolism Reveals Changes in Allocation of Nitrogen to Different Amino Acid Families When Photorespiratory Activity Changes
Source: Int J Mol Sci. 2024 Aug 1;25(15):8394. doi: 10.3390/ijms25158394 (PMC11313221; doi:10.3390/ijms25158394)
Supplement: Supplementary file 1 [file ijms-25-08394-s001.zip › Tab_S1_S2_S3.pdf]

Table S1. Shown are p-values of an ANOVA for genotype and daytime in plants grown at elevated CO2 concentration (1000 ppm). Only p-values < 0.05 are given (n=5).

Metabolite	geno	daytime	geno x daytime
Starch		<0.0001	
Ala	<0.0001	<0.0001	
PyrF	0.0330	0.0007	
Asp	0.0008	0.0267	
Ser	<0.0001	0.0152	0.0040
Gly	0.0022	0.0001	0.0179
Arg		0.0152	
Glc		<0.0001	
Frc		<0.0001	
Suc	0.0021	<0.0001	
Mal		0.0002	
Fum	0.0004	0.0026	0.0143
MF	0.0035	0.0004	0.0248
Cit	0.0073	0.0081	
NH4	0.0004	0.0175	

Table S2. Shown are p-values of an ANOVA for genotype and daytime in plants shifted from elevated CO2 concentration (1000 ppm) to ambient (ca. 450 ppm).
Only p-values < 0.05 are given (n=5).

Metabolite	geno	daytime	geno x daytime
Starch	0.0476	<0.0001	
Ala	0.0086		
Val		0.0371	
Leu			0.0466
PyrF	0.0237		
Ile		0.0479	
Asp	<0.0001	0.0055	
Thr		0.0095	
Trp			0.0094
AroF			0.0091
Ser	<0.0001	0.0040	0.0062
Gly	<0.0001	<0.0001	<0.0001
Glu	0.0061	0.0002	0.0305
Pro	0.0021		
His		0.0165	
KgF	0.0189		
Glc	0.0001	<0.0001	0.0007
Frc	0.0012	<0.0001	0.0131
Suc	0.0000	<0.0001	0.0081
Mal	0.0007	<0.0001	
Fum	<0.0001	<0.0001	
MF	<0.0001	<0.0001	
Cit	0.0027	0.0012	
NH4	<0.0001	<0.0001	0.0007

Table S3. ODE system for modeling dynamics in primary metabolites. All terms extended by _par are model parameters to be identified. All terms extended by _var are model variables that were given for certain time points. Terms carrying no extension are constants calculated from C/N ratios in metabolite pools. Because pools vary in composition for the different genotype x CO2 treatments, these constants are different for the different conditions.

```
d/dt HP     = NPS_var/6.0 + (1-alpha_par)*Gly_var*kgd_par - starchb_par*HP_var +
              starchd_par*Starch_var - ks_par*HP_var - kmf_par*HP_var - kaa1_par*HP_var +
              Ser_var*hpr_par
d/dt Starch      = starchb_par*HP_var - starchd_par*Starch_var
d/dt Sugar= (6/cSugar)*ks_par*HP_var - ex1_par*Sugar_var
d/dt MF     = (6/4)*kmf_par*HP_var - Resp_var/6.0 + alpha_par*Gly_var*kgd_par -
              kcit_var*MF_par
d/dt Cit  = (4/6)*kcit_var*MF_par - kakg_var*Cit_par + Glu_var*v0_par - Cit_var*kasf_par
d/dt AA1  = (6/cAA1)*HP_var*kaa1_par - AA1_var*ex3_par
d/dt Ser  = (Gly_var/2.0)*kgd_par - Ser_var*hpr_par - Ser_var*ex4_par - Ser_var*kshmt_par
d/dt Gly  = v0_par*(NPS_var/2.0) - kgd_par*Gly_var + Ser_var*kshmt_par
d/dt AsF  = (6/cASF)*Cit_var*kasf_par - ex5_par*AsF_var
d/dt Glu  = kglu_par*aKG_var + Gln_var*kglu_par - Glu_var*kgln_par - Glu_var*kkgf_par -
            Glu_var*v0_par
d/dt Gln  = kgln_par*Glu_var - Gln_var*kglu_par
d/dt KgF  = (5/cKGF)*kkgf_par*Glu_var - KgF_var*ex2_par
d/dt aKG  = (6/5)*Cit_var*kakg_par - kglu_par*aKG_var
d/dt NH4  = knew_par - knh4*NH4_var+Gly_var*kgd_par
```

Constants for Col-0 at elevated CO2

```
cSugar    = 8.9
cAA1      = 4.5
cAsF      = 4.2
cKgf      = 5.6
knkgf     = 0.461*kkgf_par
knasf     = (0.326*(4/4.2)*MF_var*kasf_par)/Glu_var
knaa1     = (0.247*(6/4.5)*HP_var+kaa1_par)/Glu_var
knpr      = v0_par/Glu_var
knh4      = ((knkgf+knasf+knaa1+knpr)*Glu_var)/NH4_var
```

#Constants for hpr1-1 at elevated CO2

```
cSugar    = 8.7
cAA1      = 4.6
cAsF      = 4.4
cKgf      = 5.7
knkgf     = 0.493*kkgf_par
knasf     = (0.321*(4/4.4)*MF_var*kasf_par)/Glu_var
knaa1     = (0.244*(6/4.6)*HP_var+kaa1_par)/Glu_var
knpr      = v0_par/Glu_var
knh4      = ((knkgf+knasf+knaa1+knpr)*Glu_var)/NH4_var
```

#Constants for Col-0 at ambient CO2

```
cSugar    = 9.2
cAA1      = 4.8
cAsF      = 4.2
cKgf      = 5.6
knkgf     = 0.451*kkgf_par
knasf     = (0.358*(4/4.2)*MF_var*kasf_par)/Glu_var
knaa1     = (0.244*(6/4.5)*HP_var+kaa1_par)/Glu_var
knpr      = v0_par/Glu_var
knh4      = ((knkgf+knasf+knaa1+knpr)*Glu_var)/NH4_var
```

#Constants for hpr1-1 at ambient CO2

```
cSugar     = 8.0
cAA1       = 5.0
cAsF       = 4.3
cKgf       = 5.7
knkgf      = 0.507*kkgf_par
knasf      = (0.371*(4/4.3)*MF_var*kasf_par)/Glu_var
knaa1      = (0.242*(6/5)*HP_var+kaa1_par)/Glu_var
knpr       = v0_par/Glu_var
knh4       = ((knkgf+knasf+knaa1+knpr)*Glu_var)/NH4_var
```
